# Wildervanck syndrome associated with cleft palate and short stature

**DOI:** 10.4103/0301-4738.64120

**Published:** 2010

**Authors:** Anand Kumar, Anupam Sahu, Shashikant Shetty, Vijayalakshmi P

**Affiliations:** Aravind Eye Care System 1, Anna Nagar, Madurai, Tamil Nadu-625020, India

**Keywords:** Cervico-oculo-acoustic syndrome, Duane retraction syndrome, Klippel Feil anomaly, Wildervanck syndrome

## Abstract

We report a case of Wildervanck syndrome exhibiting Klippel-Feil anomaly, Duane retraction syndrome and deafness. Since the first case was reported in 1952, there have been more reports describing this triad, either complete or incomplete. Our patient had the complete triad of the syndrome along with cleft palate and short stature. Also, a review of the literature regarding this syndrome is presented here.

Cervico-oculo-acoustic syndrome was first described by Wildervanck in 1952. The syndrome is characterized by the triad of Klippel-Feil anomaly, Duane retraction syndrome and congenital deafness.[1,2] Cases with complete as well as incomplete triad have been described in literature. This report describes a case with other associated congenital anomalies in addition to the complete triad of the syndrome.

## Case Report

A 15-year-old girl presented to our outpatient department with complaints of watering in the right eye of one week duration. She had limitation of eye movements since birth. She was born out of a second-degree consanguineous marriage, delivered normally at full term from an uncomplicated pregnancy. She had delayed developmental milestones. She had attained menarche at the age of 14 years with normal menstrual cycles and normal secondary sexual characteristics. There was no history of similar features in any of the family members.

**Figure 1:**
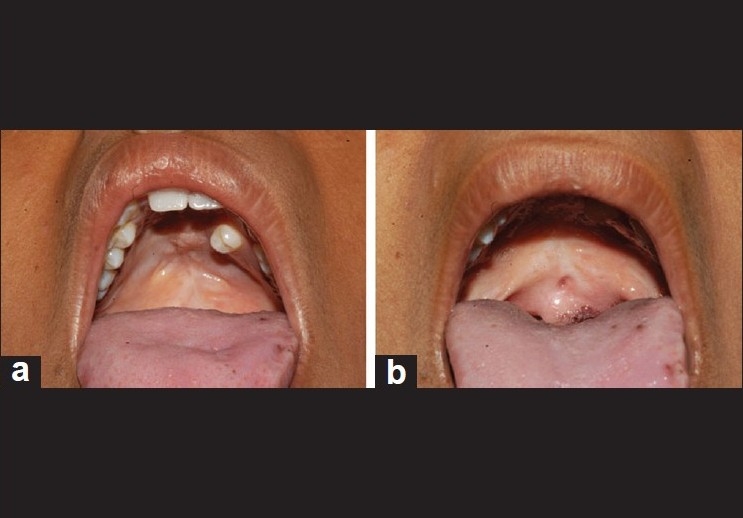
Scar of the cleft palate repair (a) and the palatal fistula (b) is seen

Cleft palate repair was done when the patient was one year old. The patient developed postoperative palatal fistula after surgery for which a revision palatoplasty was done four years back [Fig. 1].

**Figure 2:**
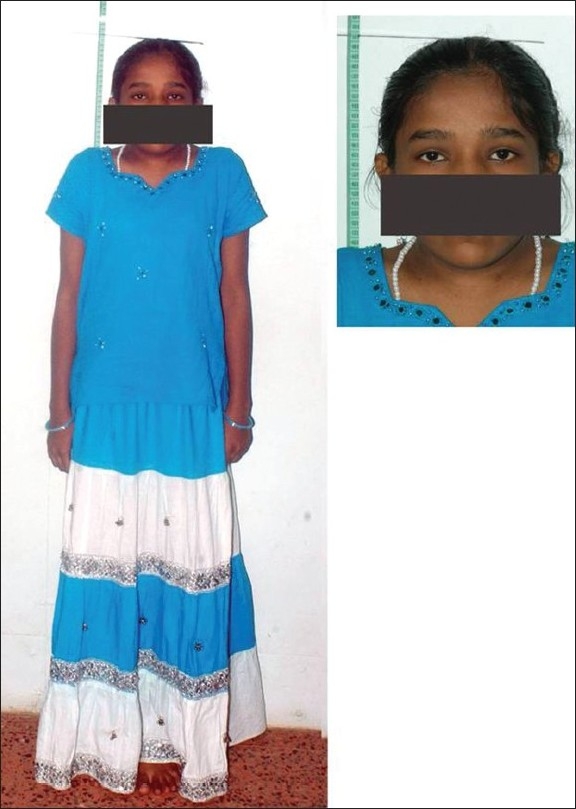
Short stature of the patient

On physical examination we found the girl to be of short stature (142 cm) with short neck and a low posterior hairline [Fig. 2]. There was no associated webbing of the neck.

Hearing and speech were impaired. Audiogram revealed a moderate sensorineural deafness. Cardiovascular and respiratory systems' examination was within normal limits.

**Figure 3:**
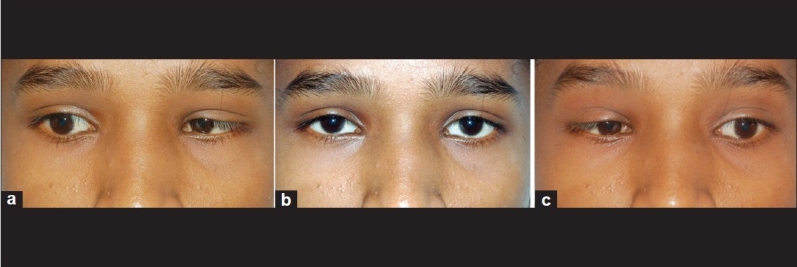
(a) Retraction of left globe on attempted right gaze (b) there is no deviation in primary position and (c) showing retraction of right globe on left gaze

Ocular examination showed normal anterior and posterior segment findings. There was no squint in the primary gaze position. However, there was limitation of abduction in both eyes with widening of palpebral fissure on attempted abduction and globe retraction on adduction, suggestive of bilateral Duane retraction syndrome. There was no upshoot or downshoot of the globe on adduction movements [Fig. 3].

**Figure 4:**
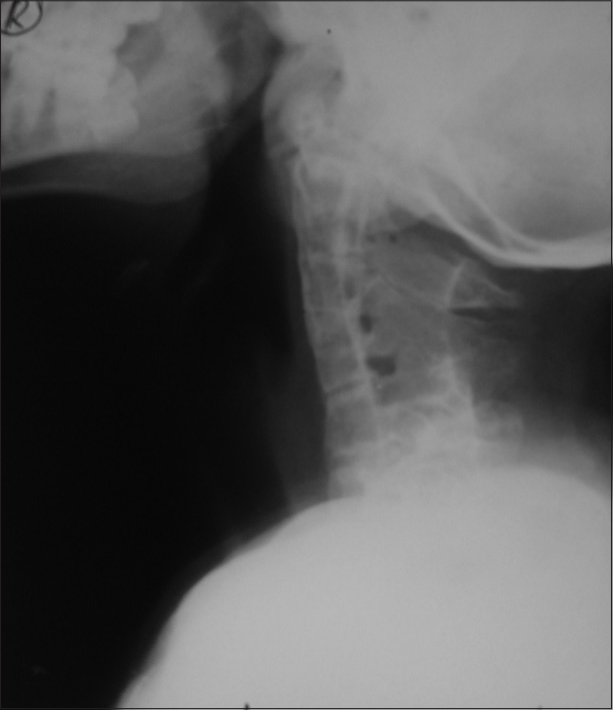
X-ray lateral view of cervical spine showing fused cervical vertebrae

Plain radiography of the cervical spine showed complete fusion of the C1, C2, and C3 cervical vertebrae with incomplete fusion of the remaining cervical vertebrae. The interspinal foramina were enlarged [Fig. 4].

The family members were examined for similar features. None of them were found to have any congenital anomalies.

## Discussion

A Medline search revealed that till date, only 45 cases of Wildervanck syndrome with a complete triad have been described.[3,4] The interesting fact is that our patient presented with additional clinical signs apart from the complete triad described by Wildervanck. These were the presence of cleft palate, short stature and delayed milestones in addition to the complete triad of the syndrome. There have been two reports each of Wildervanck syndrome being associated with cleft palate[5,6] or short stature[6,7] in isolation but till date only one case report mentions both cleft palate and short stature seen together in association with Wildervanck syndrome.[7] Our report is only the second such report with developmental delay being an additional sign found in our case.

An autosomal dominant mode of inheritance with incomplete penetrance and variable expressivity has been described in this condition.[5] A tenfold female to male preponderance has been described.[1] The cause is unknown, but Wildervanck suggests that it is due to polygenic heredity with sex limitation to the female, though McKusick raises the possibility of sex-linked dominance with fatal effect in the male.[5,6,8] Thus the anomalies described in our patient may be considered to be just coincidental or an extended part of the genetic disorder. It is important to note that the patient's family had no history of congenital anomalies.

Most of the cases described in literature have unilateral Duane retraction syndrome. Few reports have mentioned bilateral Duane retraction syndrome as one of the features of the syndrome complex.[7,8] Our patient had a bilateral Duane retraction syndrome with no deviation in the primary gaze position.

Only one-third of the patients with Wildervanck syndrome have been described as having hearing loss. Although Wildervanck stated that deafness should be sensorineural in type, cases with conductive or mixed losses have also been reported.[1,6] Audiometry in our patient revealed a moderate degree of sensorineural deafness.

Numerous other congenital anomalies have been described along with the cervico-oculo-acoustic syndrome including brainstem hypoplasia,[9] absent/ deformed auricles, severe inner ear anomalies,[10] facial asymmetry, short stature, mental retardation, cleft palate[5] and cardiac anomalies.[6] Haciyakupoglu described a patient with crocodile tears and Dandy Walker syndrome.[4] Vertebral segmentation occurs between the fourth and eighth weeks of gestation and any impairment in differentiation of the mesoderm may relate not only to cervical abnormalities but also to the combination of rare cardiovascular abnormalities.[6] In Duane retraction syndrome also, a defect occurring in the fourth week of gestation appears to be the cause, according to studies of patients prenatally exposed to thalidomide.

Although most patients of Duane retraction syndrome have only ocular features, many associated systemic defects have been observed, including Goldenhar syndrome and Wildervanck syndrome. So it becomes imperative to perform a thorough systemic examination to rule out other associated congenital anomalies.
